# Changes in Glycolytic Pathway in SARS-COV 2 Infection and Their Importance in Understanding the Severity of COVID-19

**DOI:** 10.3389/fchem.2021.685196

**Published:** 2021-09-10

**Authors:** Adalberto Fernandes Santos, Pedro Póvoa, Paulo Paixão, António Mendonça, Luís Taborda-Barata

**Affiliations:** ^1^ Faculty of Health Sciences, University of Beira Interior, Covilhã, Portugal; ^2^ CICS-UBI Health Sciences Research Centre, Universidade da Beira Interior, Lisbon, Portugal; ^3^ Department of Teaching and Research of Biochemistry, Faculty of Medicine, Agostinho Neto University, Luanda, Angola; ^4^ Polyvalent Intensive Care Unit, Centro Hospitalar de Lisboa Ocidental, Hospital de Sao Francisco Xavier, Lisbon, Portugal; ^5^ Comprehensive Health Research Center–CHRC, NOVA Medical School, Universidade Nova de Lisboa, Lisbon, Portugal; ^6^ Center for Clinical Epidemiology and Research Unit of Clinical Epidemiology, OUH Odense University Hospital, Odense, Denmark; ^7^ Laboratório de Patologia Clínica–SYNLAB, Hospital da Luz, Lisbon, Portugal; ^8^ Department of Chemistry, Faculty of Sciences, University of Beira Interior, Covilhã, Portugal; ^9^ Department of Immunoallergology, Cova da Beira University Hospital Centre, Covilhã, Portugal

**Keywords:** glycolytic pathway, COVID-19, oxidative phosphorylation, reactive oxygen species, hyperglycolysis

## Abstract

COVID-19 is an infectious disease caused by Coronavirus 2 (SARS-CoV-2) that may lead to a severe acute respiratory syndrome. Such syndrome is thought to be related, at least in part, to a dysregulation of the immune system which involves three main components: hyperactivity of the innate immune system; decreased production of type 1 Interferons (IFN) by SARS-CoV-2-infected cells, namely respiratory epithelial cells and macrophages; and decreased numbers of both CD4^+^ and particularly CD8^+^ T cells. Herein, we describe how excessive activation of the innate immune system and the need for viral replication in several cells of the infected organism promote significant alterations in cells’ energy metabolism (glucose metabolism), which may underlie the poor prognosis of the disease in severe situations. When activated, cells of the innate immune system reprogram their metabolism, and increase glucose uptake to ensure secretion of pro-inflammatory cytokines. Changes in glucose metabolism are also observed in pulmonary epithelial cells, contributing to dysregulation of cytokine synthesis and inflammation of the pulmonary epithelium. Controlling hyperglycolysis in critically ill patients may help to reduce the exaggerated production of pro-inflammatory cytokines and optimise the actions of the adaptive immune system. In this review, we suggest that the administration of non-toxic concentrations of 2-deoxy-D-glucose, the use of GLUT 1 inhibitors, of antioxidants such as vitamin C in high doses, as well as the administration of N-acetylcysteine in high doses, may be useful complementary therapeutic strategies for these patients, as suggested by some clinical trials and/ or reports. Overall, understanding changes in the glycolytic pathway associated with COVID-19 infection can help to find new forms of treatment for this disease.

## Introduction

COVID-19 is an infectious disease that emerged in December 2019 in Wuhan City, China (Zhu et al., 2020). It is caused by Coronavirus 2 Severe Acute Respiratory Syndrome (SARS-CoV-2) and, although most infected individuals are asymptomatic, severe acute respiratory syndrome may develop, and be related, at least in part, to a dysregulation of the immune system (Mehta et al., 2020; Ye et al., 2020). While still controversial in its details, such dysregulation is believed to involve at least three major components.

The first component concerns a hyperactivated state of the innate immune system, which plays a fundamental role in the progression of the disease towards more severe forms and a reserved prognosis. A similar situation was also observed in the 2002–2004 SARS and in the 2012–2015 Middle East Respiratory Syndrome (MERS) epidemics (Gralinski and Baric, 2015; Lu et al., 2020). In this context, there is production of several pro-inflammatory cytokines which induce persistent inflammation, mainly in the lung (Paces et al., 2020). Most researchers consider this cytokine dysregulation a “cytokine storm” given the higher-than-normal levels of detected cytokines (Mehta et al., 2020; Ye et al., 2020). However, although cytokines such as IL-6 are increased in COVID-19, their serum levels are clearly lower than in Acute Respiratory Distress Syndrome (ARDS) from other causes, and lower than expected in severe cases of COVID-19 (Azoulay et al., 2020; Sinha et al., 2020; Tang et al., 2020).

The second component involves a decrease, or, at least a significant delay, in production of type 1 Interferons (IFN) by SARS-CoV-2-infected cells, namely respiratory epithelial cells and macrophages (Jafarzadeh et al., 2020; Kumar, 2020; Merad and Martin, 2020). Such a deficiency in type 1 IFN is crucial to progression of COVID-19 disease since the anti-viral role of this type of interferons is essential to dealing with the infection (Jafarzadeh et al., 2020; Paces et al., 2020; Gustine and Jones, 2021).

The final component observed in more severe cases of COVID-19 involves decreased numbers of both CD4^+^ and particularly CD8^+^ T cells (Leonardi and Proença, 2020), which may also be dysfunctional and, in the latter cells, also involve hypercytotoxic states (Chen et al., 2020a; Qin et al., 2020; Schultheiss et al., 2020). In addition, decreased numbers of Natural Killer (NK) cells in peripheral blood can also be observed, which further weakens anti-viral responses, since CD8^+^ T cells and NK cells are the main drivers of anti-viral cellular immune responses (Merad and Martin, 2020; Paces et al., 2020).

This mini-review takes into account the three components mentioned above and will aim at putting forward the concept that changes in the glycolytic pathway that occur during SARS-CoV-2 infection significantly contribute to the immune dysregulation observed in severe COVID-19 respiratory infections and also that specific drug treatments that interfere with such changes in the glycolytic pathway may be clinically beneficial in infected patients.

## The Glycolytic Pathway

**FIGURE 1:**
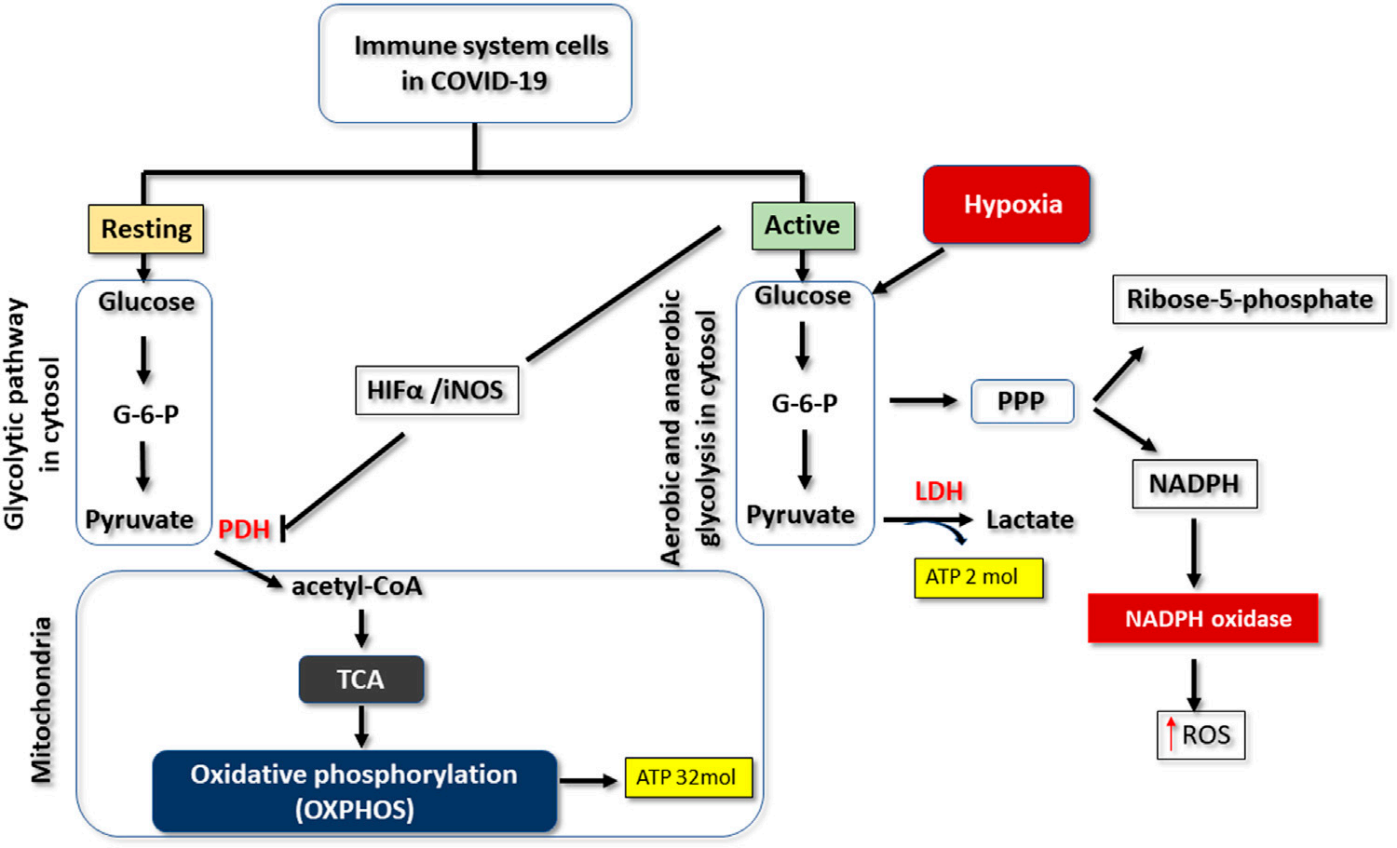
Changes that can occur in the glycolytic pathway in COVID-19. When immune cells are resting, glucose is metabolised *via* the glycolytic pathway, yielding pyruvate as a product. This substrate is subsequently used in the tricarboxylic acid cycle (TCA) to form intermediates that will be processed by oxidative phosphorylation to produce 32 molecules of ATP, for each molecule of glucose. When immune cells are activated or hypoxic, glucose metabolism is reprogrammed. There is an increase in the type 1 alpha hypoxia-inducing factor (HIFa), which inhibits pyruvate dehydrogenase enzyme complex (PDH). Pyruvate is thus transformed into lactate by the action of lactate dehydrogenase (LDH). But this mechanism motivates the capture of more glucose for the synthesis of further ATP molecules. There is also activation of the pentose phosphate (PPP) pathway that produces ribose and NADPH. The latter can be used by immune cells through the actions of NADPH oxidase to produce reactive oxygen species (ROS).

The glycolytic pathway is a metabolic pathway of foremost importance in supporting life in almost all organisms (aerobic or anaerobic). It occurs in the cytosol and, under aerobic conditions, aims to convert the active form of glucose (glucose-6-phosphate) (C_6_H_11_O_9_P) into pyruvate (C_3_H_4_O_3_) (Li et al., 2018) (Figure 1). In this pathway, there is cleavage of glucose (C_12_O_6_H_12_), resulting in the production of potentially energetic intermediates such as pyruvate and NADH (C_21_H_29_N_7_O_14_P_2_), as shown in the following equation “C_6_ H_12_ O_6_ + 2ADP + 2Pi+ 2NAD + → 2C_3_ H_4_ O_3_ + 2ATP + 2NADH + 2H^+^” (Chen et al., 2018; Dunn et al., 2016). These intermediates are subsequently used in the mitochondria, in the tricarboxylic acid (TCA) cycle for the cell respiration process and oxidative phosphorylation (OXPHOS), which in turn produces other intermediates that are used in the electron transport chain for production of large amounts of adenosine triphosphate (ATP) (C_10_H_16_N_5_O_13_P_3_), which is the most important form of chemical energy for the cell (and for life) (Figure 1) (Chen et al., 2018). At the end of the glycolytic pathway, only two molecules of ATP are produced per glucose molecule, whereas through OXPHOS the yield rises to 32 molecules of ATP per glucose molecule (Ostroukhova et al., 2012; Dunn et al., 2016). Under aerobic conditions, the pyruvate produced in the glycolytic pathway is transported into the mitochondria and by the action of the pyruvate dehydrogenase enzyme complex, it is converted into acetyl CoA (C_23_H_38_N_7_O_17_P_3_S), which is used in oxidative phosphorylation to produce important intermediates for the electron transport chain (Dunn et al., 2016). Oxygen is used as the final electron acceptor at the end of the electron transport chain, and part of this oxygen is transformed into reactive oxygen species (ROS) by mitochondria, which are the main generators of ROS, even in a physiological context (Ostroukhova et al., 2012).

In hypoxia situations, changes occur in the energy metabolism of cells in general, due to the lack or decrease of oxygen supply. In particular, pyruvate that was converted to acetyl CoA is now reduced into lactate (C_3_H_5_O_3_) by the action of the lactate dehydrogenase (LDH) enzyme (Figure 1) (Ostroukhova et al., 2012). This means that the production of ATP by OXPHOS is compromised (Ostroukhova et al., 2012), and the decrease in intracellular oxygen supply reduces the production of ATP by the electron transport chain. Thus, in hypoxia, cells can only rely on the glycolytic pathway, which produces just two molecules of ATP per glucose molecule, a situation that explains the greater consumption of glucose (hyperglycolysis) as the main source of energy production (Dunn et al., 2016). It should be highlighted that hyperglycolysis, that occurs in such cellular hypoxia situations, is due to reprogramming in the metabolism of glycolysis, and is characterised both by the production of ATP exclusively *via* the glycolytic pathway, and by inhibition of oxidative phosphorylation (Figure 1) (Li and Zhang, 2016). Hyperglycolysis or metabolic reprogramming may also occur in normoxic situations, namely when there is a need for the immediate use of glucose by cells, in order to increase their performance in carrying out tasks such as proliferation, activation, or synthesis of molecules. This strategy is known as aerobic glycolysis, since the increase in glucose consumption happens in the presence of oxygen (Li and Zhang, 2016).

In the situations involving metabolic reprogramming described above, there is thus an *enzymatic redefinition* that involves decreased pyruvate dehydrogenase activity and increased lactate dehydrogenase (LDH) levels (Figure 1) (Seheult et al., 2017). In such metabolic reprogramming, increased expression of hypoxia-1 alpha-inducing factor (HIF-1α) protein contributes to the previously mentioned decreased activity of pyruvate dehydrogenase by inhibiting the pyruvate dehydrogenase complex (Andrejeva and Rathmell, 2017; Kumar, 2020). This explains the inhibition of oxidative phosphorylation, giving rise to aerobic glycolysis or hyperglycolysis (Figure 1) (Bergsneider et al., 1997; Andrejeva and Rathmell, 2017; Kumar, 2020).

In inflammatory and septic conditions, as happens in a patient with severe COVID-19, there is also reprogramming of glucose metabolism, associated with increased expression of the ubiquitous sodium-independent glucose transporter 1 (GLUT1) in immune and non-immune cells, increased expression of glycolytic enzymes, such as phosphofructokinase-2, as well as inhibition of pyruvate dehydrogenase enzyme complex by increasing the expression of HIF-1α, which contributes to the increase in glucose uptake (Icard et al., 2021). These hyperglycolytic events that occur in response to stress (i.e., infection-induced stress) also contribute to viral replication and worsening of inflammation (Kumar, 2020). Thus, the inhibition of aerobic hyperglycolysis in SARS-COV-2-infected cells may help to control viral replication and decrease inflammation, as has been shown in some *in vitro* studies in which nontoxic concentrations of 2-deoxy-d-glucose inhibited viral replication in human, colon carcinoma-derived Caco-2 cells (Bojkova et al., 2020).

## Increased Glucose Consumption by Cells in SARS-COV-2 Infection

**FIGURE 2:**
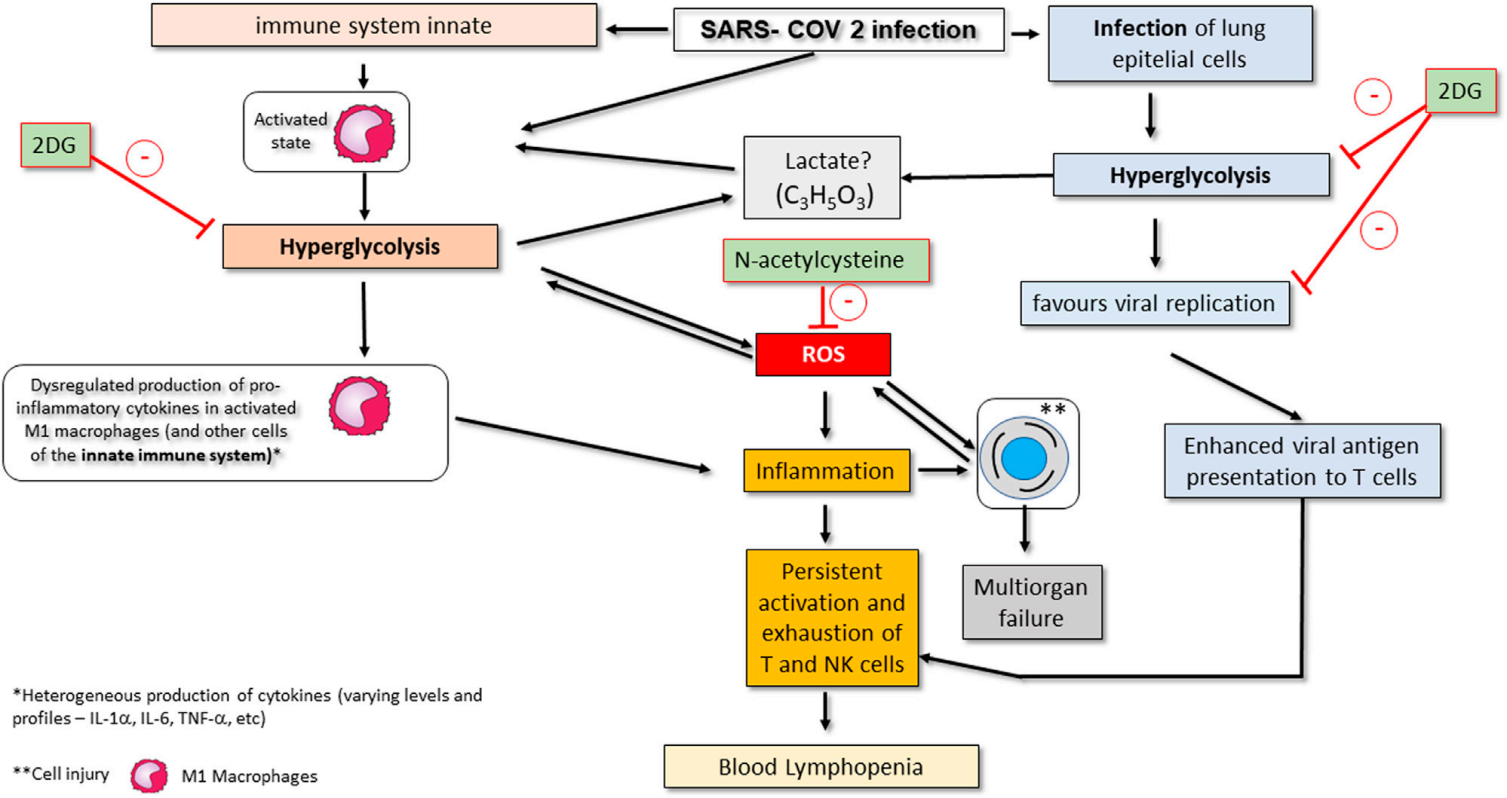
Conceptual model of SARS-CoV-2-associated changes in the glycolytic pathway and its consequences on COVID-19 in critically ill patients. Viral infection activates the innate immune system, namely M1 macrophages. Activation of macrophages promotes metabolic changes for their effective action, including an increase in consumption of glycolysis (hyperglycolysis) and a change in the strategy for ATP synthesis (*metabolic reprogramming*). These changes favour the production of ROS and worsen inflammation. Hyperglycolysis may also promote an increase in serum lactate. SARS-CoV-2 infection also affects epithelial lung cells by making them increase their glucose consumption, which makes viral replication viable. Infected epithelial cells also show enhanced presentation of viral antigens to T lymphocytes (particularly CD8^+^ T cells), and NK cells are also affected, with both cell types becoming more chronically activated. These events promote exhaustion and subsequent apoptosis of T and NK cells, thereby contributing to peripheral lymphopenia. Drugs that may eventually be used as complementary treatment of severe COVID-19 infection by inhibition infection-associated hyperglycolysis include 2-deoxy-d-glucose (2DG), N-acetylcysteine or GLUT1 inhibitors. Administration of 2DG inhibits hyperglycolysis in innate immune system cells such as macrophages; on the other hand, the administration of N -acetylcysteine inhibits the increase in ROS production by activating glutathione (see therapeutic strategies section). The action of GLUT1 inhibitors is not shown but involves blocking glucose uptake in activated and infected immune system and epithelial cells.

SARS-CoV-2 binds *via* its spike (S) protein to receptors for the angiotensin-converting enzyme 2 (ACE2) on target cells, and this protein: receptor complex is internalised by cells, namely respiratory epithelial or innate immune system cells (Zhang et al., 2020a). In infected cells, SARS-CoV-2 can induce a set of changes at various levels, namely considerable changes in the glycolytic pathway (Ding et al., 2017; Alfano et al., 2021) (Figure 2). Various mechanisms contribute to this.

First, once inside a cell, the presence of SARS-CoV-2 activates mechanisms, namely mitochondrial oxidative damage, that upregulate intracellular production of ROS, which, in turn, increase cellular injury and cause intracellular stress thereby forcing the infected cell to have increased glucose concentrations. Thus, these cells reprogram their glucose metabolism, and this may involve changes in the final product of the glycolytic pathway (production of lactate instead of pyruvate), activation and inhibition of enzymes (activation of lactate dehydrogenase, and inhibition of pyruvate dehydrogenase) that favour the infectious scenario (Saleh et al., 2020). COVID-19 infection-associated intracellular stress increases endoplasmic reticulum (ER) stress which has been found to be associated with increased expression of Glucose-Regulated Protein 78 (GRP78) at the membrane level, in various settings (e.g., cancer, obesity) (Pfaffenbach and Lee, 2011). Increased levels of GRP78 are not only important for reducing ER oxidative stress but translocation of this protein to the membrane also aims to meet the increased demand for glucose inside the infected and stressed cell, since GRP78 contributes to increased glucose uptake into cells (Madhavan and Nagarajan, 2020). In fact, elevated GRP78 levels are significantly higher in COVID-19 infected patients with or without pneumonia than in healthy controls and also tend to be higher than in COVID-negative patients with pneumonia (Sabirli et al., 2021).

Second, patients with severe SARS-CoV-2 infection have been shown to have higher levels of lactate dehydrogenase (LDH) in the peripheral blood compared to patients with mild or moderate disease (Chen et al., 2020b; Mo et al., 2020; Tian et al., 2020). For this reason, LDH is used as one of several relevant parameters to assess the severity of COVID-19 (Zhang et al., 2020b). The augmented levels of LDH in COVID-19 are related to an increase in the production of lactate, which means that changes in the glycolytic pathway and/or low oxygen conditions occur in cells in the context of infection and/or activation (Ding et al., 2017; Strachecka et al., 2019; Farhana and Lappin, 2020; Morassi et al., 2020). In SARS-CoV 2 infection, with abrupt lung involvement, there is a decrease in oxygen supply in lung cells due to inflammatory events resulting from the infection (Jahani et al., 2020; Serebrovska et al., 2020; Ye et al., 2020) This situation leads to a set of changes in the glycolytic pathway, as well as increased glucose uptake, that impair cell function, thereby worsening events that eventually lead to multiorgan dysfunction, as increased glucose uptake promotes ideal conditions for an inflammatory environment and ROS production (Paces et al., 2020).

## Consequences of Glucose Metabolism Reprogramming in Infected Lung Epithelial Cells

Pulmonary epithelial cells infected by SARS-COV 2 also have an increased expression of HIF-1 a which is closely associated with metabolic reprogramming (Kumar, 2020). In these cells, metabolic reprogramming increases consumption of glucose (Figure 2) and as a consequence, it augments local secretion of proinflammatory cytokines (Neufeldt et al., 2020) that are responsible for the infiltration of monocytes and neutrophils into the lungs. On the other hand, the virus inhibits the secretion of type 1 IFN in human airway epithelial cells (Vanderheiden et al., 2020), which reduces antiviral mechanisms and may also contribute to worsening of the inflammatory and septic condition (Kumar, 2020).

In general, in severe situations of SARS-CoV infection, metabolic reprogramming in human airway epithelial cells promotes increased glucose uptake and increased oxidative stress that culminates in increased lactate production, and mitochondrial damage, as well as dysregulation of the innate immune system (including overactivation and functional dysregulation of the innate immune system, as well as functional depletion and dysregulation of T cells), which results in a poor prognosis in critically ill patients (Li et al., 2018).

## Changes in the Glycolytic Pathway in Immune Cells in SARS-CoV-2 Infection

Some *in vitro* studies have shown that cultures of SARS-CoV-2-infected bronchoalveolar lavage monocytes from patients with COVID-19 have upregulated expression of genes associated with glycolysis (Codo et al., 2020). In addition, the same study also showed that SARS-CoV-2 infection, but not infection with the human H1N1 influenza A or respiratory syncytial virus, specifically stimulated glycolysis and increased glycolytic capacity and glycolytic reserve in monocytes (Codo et al., 2020).

The production of cytokines in immune cells such as macrophages, dendritic cells or neutrophils, as well as activation of these cells is intrinsically linked to reprogramming of energy metabolism (Figure 2) (Patel et al., 2019; Codo et al., 2020), and this also occurs in SARS-CoV-2 infection (Kumar, 2020). Activated macrophages undergo metabolic reprogramming, by switching from oxidative phosphorylation to aerobic glycolysis, a strategy that proves to be more profitable and effective for ATP production required for the secretion of cytokines and all other effector actions that these cells need to perform in the case of infection, namely antigen presentation to T cells (Icard et al., 2021).

Thus, SARS-CoV-2 infection activates the innate immune system and, if this process becomes uncontrolled, it can result in the dysregulation of cytokine production (Figure 2), involving increased production of pro-inflammatory cytokines, namely interleukin-6 (IL-6), IL-8, TNF- a and others, which contribute to the development of ARDS (Fu et al., 2020). In the context of ARDS it is accepted that there is a decrease in tissue oxygen which, if severe, is reflected in cellular hypoxia, thereby further contributing to metabolic reprogramming in immune cells (Jahani et al., 2020; Serebrovska et al., 2020).

However, in some situations, the alteration in the glycolytic pathway does not occur towards production of aerobic glucose, which induces a more efficient immune response, but rather towards oxidative phosphorylation, which compromises and delays this response. In this sense, the virus inhibits molecular signaling mechanisms that activate aerobic glycolysis, thereby impairing the synthesis of type 1 IFN and contributing towards dysregulated production of pro-inflammatory cytokines, a scenario observed not only in infected epithelial cells, but also in infected dendritic cells and peripheral blood mononuclear cells (Gardinassi et al., 2020).

Furthermore, persistent inflammation mediated by the innate immune system, namely M1 macrophages, as well as the constant presentation of viral antigens to CD8^+^ cytotoxic T cells contribute to the exhaustion of the latter cells (Figure 2), and, as consequence, their energy metabolism also changes from aerobic glucose to oxidative phosphorylation (Fenwick et al., 2019; Kumar, 2020). In fact, in infected cytotoxic CD8^+^ T cells, the production of ATP ends up being provided by oxidative phosphorylation, in contrast to aerobic glycolysis. This situation is detrimental to infected cells due to the fact that they produce more ROS *via* mitochondria, may become more dysfunctional and enter apoptosis (Fenwick et al., 2019).

## Will There be Lactic Acidosis in SARS-CoV-2 Infection Due to Alteration of the Glycolytic Pathway?

Changes in glucose metabolism that promote hyperglycolysis lead to the formation of significant amounts of lactate (Figure 1) (Brooks, 2018). Lactate production can have differential effects on immune cells, namely indirect inhibition of production of type 1 IFN in macrophages (Kumar, 2020; Zhang et al., 2019). These effects would also be expected in patients with SARS-CoV-2 infection, because of the changes in the glycolytic pathway that promote overactivation of the innate immune system (Figure 2) (Fenwick et al., 2019; Ye et al., 2020). High levels of lactate could also be expected due to pulmonary impairment in critically ill patients with SARS-CoV-2. However, lactate does not seem to be associated with a poor prognosis and progression of the disease, since its serum levels are not increased in the overwhelming majority of critically ill patients with COVID-19 (Mo et al., 2020; Tang et al., 2020). Recently, a retrospective study that aimed to identify acid-base disorders in patients with COVID-19 identified only one patient whose metabolic acidosis was caused by a slight increase in peripheral blood lactate, in a sample of 211 patients (Alfano et al., 2021). In fact, the vast majority of studies do not report such a condition in patients with severe COVID-19 infection who are hospitalized in ICU wards, even when LDH levels are increased (Zhang et al., 2019). This is an interesting situation that could lead to a discussion about the effects of hypoxia and hyperglycolysis in COVID-19 from a metabolic point of view, and raise the question of why there is no detectable lactic acidosis in the peripheral blood of critically ill patients with COVID-19. The mechanism behind the lack of an increase in lactate levels is not known, but one hypothesis may be that produced lactate can be rapidly consumed by the lactate-producing cell as it is used, for instance, for binding to mitochondrial antiviral-signaling protein (MAVS) which may result in decreased type 1 IFN production as was seen in HEK-293 human kidney cell lines (Zhang et al., 2019). On the other hand, there is also the possibility that the non-accumulation of lactate in the bloodstream may be due to its consumption by the liver and kidneys, through the Cori cycle, allowing the production of more glucose. The increase in LDH could reinforce this idea, since this enzyme is also involved in the conversion of lactate to pyruvate in the Cori cycle (Marik and Bellomo, 2013; Gurung and Jialal, 2020; Levitt et al., 2020; Yang et al., 2020).

## Changes in the Glycolytic Pathway Promote Reactive Oxygen Species Production in SARS-CoV-2 Infection

As was previously mentioned, hyperglycolysis leads to overproduction of ROS (Figure 2). Several cells in different organs, as well as macrophages and neutrophils have an enzyme complex called NADPH oxidase (Figure 1) (Baillet et al., 2017) Among other functions, this transmembrane complex produces ROS, thereby acting as a relevant source of such oxygen radicals in infectious processes (Baillet et al., 2017). NADPH oxidase uses NADPH produced in the pentose phosphate pathway (PPP) as an electron donor for the production of ROS (Figure 1) (Andrejeva and Rathmell, 2017; Baillet et al., 2017). As hyperglycolysis promotes an increase in the production of glucose-6-phosphate, an intermediate of the glycolytic pathway that is used in the pentose phosphate pathway for the production of ribose-5-phosphate and NADPH, there will necessarily be an increase in ROS production by NADPH oxidase (Figure 1) (Baillet et al., 2017).

The increase in ROS by activating NADPH oxidase further stimulates hyperglycolysis to produce more ATP, and the production of ATP in turn further activates NADPH oxidase. ATP is used to phosphorylate the components of NADPH oxidase, thus leading to its activation. Therefore, the assembly of the enzyme complex is improved to increase production of ROS (Baillet et al., 2017). In addition, there is a process of mutual activation between hyperglycolysis and NADPH oxidase that is mediated by phosphofrutokinase-2 (Batushansky et al., 2019).

It is generally accepted that these situations of hyperglycosis in COVID-19 infection augment the activation of the immune system and worsen inflammatory response, namely *via* ROS production (Saleh et al., 2020; Icard et al., 2021). Thus, since there is a considerable increase in ROS in severe COVID-19, and little or no production of natural antioxidants by the body, the use of antioxidants could be part of the therapeutic protocols of critically ill patients with COVID-19. In fact, an epidemiological analysis has recently suggested that an anti-oxidant-rich dietary approach may enhance Nuclear factor (erythroid-derived 2)-like 2 (Nrf-2)-associated antioxidant effect, thereby contributing to mitigation of the severity of COVID-19 infection (Bousquet et al., 2020; Bousquet et al., 2021).

## Therapeutic Strategies

Increased glucose consumption (hyperglycolysis), as part of metabolic reprogramming in which there is enzymatic redefinition, appears to be the basis for severity and poor prognosis in critically ill patients infected with SARS-COV-2. Thus, controlling hyperglycolysis in critically ill patients would help to control the dysregulated or exaggerated production of pro-inflammatory cytokines and optimise the action of the adaptive immune system. In this context, the administration of non-toxic concentrations of 2-deoxy-d-glucose (2-DG) may be a strategy for minimizing events resulting from increased glucose consumption. 2-DG is a glucose analogue which inhibits the glycolytic pathway, through the inhibition of phosphoglucoisomerase. Thus, there will be no formation of glucose-6-phosphate, which is a crucial intermediate for the continuity of the glycolytic pathway (Saleh et al., 2020; Verma et al., 2020). This mode of action makes 2-DG a possibly useful drug for inhibition of metabolic reprograming which also occurs in cancer, resulting in decreased glucose consumption and inhibition of the glycolytic pathway in cancer cells (Woodward and Cramer, 1952). Studies carried out *in vitro* and in murine models showed that 2-DG may induce antitumor CD8^+^ T cell-mediated cytotoxicity and increased production of IFN-g (Sasaki et al., 2021). In addition, 2-DG in association with radiotherapy also has other immune effects by increasing cell membrane expression of molecules that are relevant to antigen presentation (MHC II and CD86) by dendritic cells and reducing production of the pro-inflammatory cytokine TNFα (Farooque et al., 2014). All of these actions may be beneficial in the context of severe COVID-19 infection. However, evidence of clinical benefit is warranted in this context. Although 2-DG monotherapy may need to involve high doses and toxicity, small phase I and phase II clinical trials in patients with glioblastoma showed that combined treatment of 2-DG and radiotherapy was well tolerated, moderately improved survival, and improved quality of life (Mohanti et al., 1996; Singh et al., 2005), although there were problems with study design and definition of endpoints in these studies (Mohanti et al., 1996; Singh et al., 2005; Halder and Mehta, 2021). Thus, various aspects regarding safety and efficacy of 2-DG as complementary treatment both in cancer and in infectious diseases such as COVID-19 still have to be addressed by robust, randomized, double-blind, placebo-controlled phase III clinical trials. Currently, at least one phase II clinical trial aiming at studying safety and efficacy of 2-DG in COVID-19 patients is underway (CTRI/2020/06/025,664; Cochrane Central register of Controlled Trials).

On the other hand, pharmacological strategies such as the use of GLUT 1 inhibitors (GLUT-1i) aimed at inhibiting glucose transporters could be evaluated in the treatment of critically ill patients, since there is an increase in the expression of GLUTs in immune and non-immune cells in these patients. In fact, the use of glucose inhibitors as a therapeutic strategy in other infectious diseases (e.g., Zika virus or Human T cell Leukemia Virus infections) and in various types of cancer cells has been documented *in vitro* by other authors (Manel et al., 2003; Dominguez Rieg and Rieg, 2019; Lin et al., 2019; Reckzeh and Waldmann, 2020; Cao et al., 2021) but data are necessary in the context of the possible use of GLUT-1i as complementary therapy in severe COVID-19 infection.

Changes in the glycolytic pathway in critically ill, SARS-COV-2-infected patients lead to increased production of ROS and a decrease in compounds that control such radicals, such as glutathione. Based on this scenario, the use of antioxidants such as vitamin C in high doses as a therapeutic adjuvant in critically ill patients with COVID-19 has been shown to be a promising strategy. A prospective, randomised double-blind clinical trial with a sample of 308 critically ill COVID-19 patients, demonstrated that the use of high doses of vitamin C improved lung function and reduced mortality from multiple organ failure. It has been shown, despite the need for further studies, that this strategy would help to reduce ARDS events and multiorgan damage caused by excess ROS from alterations in the glycolytic pathway (Liu et al., 2020).

On the other hand, still with the perspective of mitigating ROS-induced events, another interesting therapeutic approach would be the administration of N-acetylcysteine in high doses. This compound is a reduced glutathione (GSH) intermediate (Suhail et al., 2020). Its use aims to increase GSH (a natural antioxidant) levels in the body, since such levels are decreased in critically ill COVID-19 patients (Bartolini et al., 2021; Karkhanei et al., 2021). Beneficial effects of N-acetylcysteine have been demonstrated in clinical trials in various infections, including influenza and influenza-like infections, namely H1N1 influenza pneumonia or even community acquired pneumonia (De Flora et al., 1997; Zhang et al., 2018). In COVID-19, administration of N-acetylcyteine, alone or together with other types of supplementation may be beneficial in patients with different degrees of severity of SARS-CoV-2 infection, as suggested by a few small-sized, non-controlled clinical interventional studies or case reports (De Flora et al., 2020; Avdeev et al., 2021; Wong et al., 2021), although these results may not apply to patients with SARS-CoV-2-induced ARDS (de Alencar et al., 2021; Taher et al., 2021), and still need to be more robustly analysed in larger, randomised, placebo-controlled studies.

## Conceptual Model

A general conceptual model of all COVID-19-associated changes in glycolytic pathways as well as potential points for therapeutic interventions is shown in Figure 2.

## Conclusion

Events related to changes in the glycolytic pathway in COVID-19 infection may explain the underlying pathology and severity of some cases of the disease. However, although there is clear scientific evidence showing changes in glycolysis in COVID-19 infection, there is still little evidence linking such changes to worse prognosis of COVID-19. Thus, the current review clearly shows that such an association should be considered in future research in this context.

Furthermore, there seems to be a relationship between hyperglycolysis and dysregulation of the immune system, as well as cellular damage. However, further studies are needed to consolidate these ideas and, perhaps, to find therapeutic targets that can mitigate the deleterious events that arise from this context.

Since there is a considerable increase in ROS in severe COVID-19, and little or no production of natural antioxidants by the body, we think that perhaps the use of antioxidants could be part of the therapeutic protocols of critically ill patients with COVID-19.
